# Study on Water Resource Carrying Capacity and Crop Structure Optimization Based on Gray Relational Analysis

**DOI:** 10.3390/plants14050685

**Published:** 2025-02-23

**Authors:** Lingyun Xu, Bing Xu, Ruizhong Gao, Guoshuai Wang, Delong Tian, Yuchao Chen, Jie Zhou, Xiangyang Miao, Pingxia Wang

**Affiliations:** 1 College of Water Conservancy and Civil Engineering, Inner Mongolia Agricultural University, Hohhot 010018, China; lingyunxu0012@163.com (L.X.); chenyuchao132@163.com (Y.C.); zhoujiepiaoliang@163.com (J.Z.); 2 Institute of Water Resources for Pastoral Area Ministry of Water Resources, Hohhot 010020, China; imau_wgs@163.com (G.W.); mkstdl@126.com (D.T.); miaoxiangyang1108@163.com (X.M.); 3 Yinshanbeilu Grassland Eco-Hydrology National Observation and Research Station, China Institute of Water Resources and Hydropower Research, Beijing 100038, China; 4 Autonomous Region Collaborative Innovation Center for Integrated Management of Water Resources and Water Environment in the Inner Mongolia Reaches of the Yellow River, Hohhot 010018, China; 5 Inner Mongolia Key Laboratory of Ecohydrology and High-Efficient Utilization of Water Resources, College of Water Conservancy and Civil Engineering, Inner Mongolia Agricultural University, Hohhot 010018, China; 6 Inner Mongolia Mechanical and Electrical Vocational Technical College, Hohhot 010070, China; bingxue0798@163.com

**Keywords:** water resource carrying capacity, gray relational degree, planting structure optimization, machine learning

## Abstract

This study addresses challenges such as insufficient irrigation water quotas, severe groundwater over-extraction, and conflicts around crop water usage within the mixed-cropping areas of the Inner Mongolia Yellow River Basin. Five evaluation factors—water resource utilization efficiency, irrigation rate, degree of development and utilization, supply modulus, and demand modulus—were selected for a gray relational analysis to assess the 2023 water resource carrying capacity. A crop structure optimization model was developed using machine learning, focusing on minimizing water use while maximizing economic benefits. The results indicate that groundwater resources are nearing critical levels, with many regions showing low carrying capacities and supply–demand conflicts. Key issues include unreasonable planting structures and excessive irrigation quotas, leading to significant water waste. To optimize resource utilization, it is recommended to reduce the food crop planting area by 0.0194 × 10^4^ hm^2^ and increase economic and forage crops by 0.0106 × 10^4^ hm^2^ and 0.0116 × 10^4^ hm^2^, respectively. This adjustment would lead to a total water utilization reduction of 0.0289 × 10^6^ m^3^ per year, an increase in total yield of 4340.86 tons, and an increase in total economic benefit of CNY 6,559,200, thus leading the cropping structure towards greater rationality. The findings provide valuable insights for optimal water resource allocation in mixed-cropping irrigation areas.

## 1. Introduction

Water resources are fundamental for maintaining the ecological balance of the Earth and promoting social development [1]. The Yellow River Basin in Inner Mongolia is an important grain production base in China [2] and also serves as an important forage base. Within the limited water resources available, agricultural water usage in the Yellow River Basin accounts for over 70% of the total water consumption in national river basins [3]. In recent years, with the continuous expansion of irrigated areas, the degree of surface water resource development in most regions of the Inner Mongolia Yellow River Basin has exceeded its limits. Currently, due to insufficient irrigation water quotas from the Yellow River, mixed-cropping areas of grains, economic crops, and forages have resorted to groundwater irrigation, leading to unclear assessments of groundwater resource carrying capacity and raising concerns about the rationality of the planting structure.

Thus, exploring the carrying capacity of groundwater resources and the crop structure in these mixed-cropping areas is crucial for reducing water resource consumption given the limited availability of water. Evaluating water resource carrying capacity can effectively clarify the state of water resources and propose reasonable protective measures for water resource issues. Various methods exist for evaluating water resource carrying capacity, such as conventional trend analysis, fuzzy comprehensive evaluation, principal component analysis, system dynamics, and gray relational analysis [4,5,6,7]. For instance, He [8] established a model for optimizing planting structure and regulating water quantity and quality in the Yanghe Basin based on Dempster–Shafer theory and fuzzy comprehensive evaluation theory, demonstrating that a rational optimization scheme for watershed water resources can enhance the carrying capacity. Zhang [9] developed a water ecological carrying capacity (WECC) evaluation index system and a system dynamic simulation model for the Siping region using system dynamics (SDs) and an analytic hierarchy process (AHP) to assess water ecology and carrying capacity. Duan [10] employed 30 quantitative indicators to establish a driver–pressure–state–impact–response (DPSIR) model integrated with Tapio decoupling analysis in the Chaohu Basin, indicating that strengthened watershed management could lead to substantial improvements in water quality. Wang [11], based on principal component analysis and system dynamics, proposed an evaluation system for water resource carrying capacity in Qingyang city, believing that the pressure of water resources could be alleviated by adopting a two-way adjustment strategy of supply and demand, providing a reference for the regulation of water resources in Qingyang city. Zhang [12] proposed the evaluation of the water resource carrying capacity of the Anhui province by gray correlation analysis and an evaluation model. The results showed that the water resource carrying capacity of each prefecture-level city was a grade II critical load. Guan [13] utilized a CRITIC-TOPSIS-gray relational evaluation model to assess the water resource utilization efficiency in the Huai River Basin, revealing low water utilization rates in Jiangsu but an overall upward trend in the basin. Wang [14] proposed the use of a non-parametric test and gray correlation analysis to study the correlation between various influencing factors and groundwater depth in the Fenhe River Basin, so as to grasp the dynamic development law of groundwater in the Fenhe River Basin under this new situation and improve the protection and management level of groundwater resources. Chen [15] evaluated the carrying capacity of land and water resources in the Jiaokou irrigation area using indicators of soil and water resources, socio-economics, and ecological environment, applying the TOPSIS model. The results indicated that enhancing the irrigation area’s carrying capacity requires a focus on agricultural development and ecological protection. A rational planting structure is essential for optimizing the allocation of land and water resources in irrigation areas. Li [16] developed a planting structure optimization model based on water-saving irrigation technologies in the Shihezi irrigation area, showing that a proportion of 0.84 or 0.82 membrane drip irrigation technology favored sustainable development in the area. Wu [17] established a multi-objective optimization model for a crop planting structure on the southern bank of the Xiaolangdi Reservoir irrigation area using the GWO algorithm, indicating that the optimization results from the planting structure and cooperative game strategies outperformed other schemes. Wang [18] constructed a multi-objective crop planting structure optimization model based on NSGA-II with constraints of land and water resources and social demands, aiming to maximize economic and ecological benefits while minimizing total irrigation water consumption. The results suggest that, in plain areas under high water-saving requirements, the proportion of vegetable and fruit planting should be reduced, while in mountainous areas, the area for fruit trees should be expanded and the grain area minimized.

In summary, the evaluation of water resource carrying capacity and the optimization of crop structures play a crucial role in the sustainable development of irrigation areas. Due to significant groundwater extraction in these areas, the carrying capacity of groundwater resources remains unclear, raising questions about the rationality of the planting structure. Therefore, this study focused on the mixed-cropping areas of grains, economic crops, and forages in the Inner Mongolia Yellow River Basin. It constructs a water resource carrying capacity evaluation model based on gray relational analysis and a crop structure optimization model using machine learning, aiming to clarify the region’s water resource carrying capacity and identify the optimal planting ratios. The objective of this research was to provide a foundational framework for the optimal allocation of water resources in mixed-cropping irrigation areas and serve as a reference for their sustainable development.

## 2. Results

### 2.1. Analysis of Water Resources and Planting Structure in the Irrigation Area

#### 2.1.1. Analysis of Water Resources in the Irrigation Area

Based on reports and field investigations, the calculated area of Tumotey Right Banner is 2769 km^2^. The total water resource per year is 440 × 10^6^ m^3^, comprising 163 × 10^6^ m^3^ from surface water and 277 × 10^6^ m^3^ from groundwater. The available water volume per year is 295 × 10^6^ m^3^, with surface water accounting for 141 × 10^6^ m^3^ and groundwater 154 × 10^6^ m^3^. The total water consumption indicator per year is 278 × 10^6^ m^3^, with agricultural water consumption at 20.691 × 10^6^ m^3^. The calculated area for the irrigation zone is 71.92 km^2^. The total water resource in this area per year is 17.6479 × 10^6^ m^3^, including 6.5377 × 10^6^ m^3^ from surface water and 11.1102 × 10^6^ m^3^ from groundwater. The total agricultural water resource per year is 15.2045 × 10^6^ m^3^, with actual agricultural water usage at 12.7733 × 10^6^ m^3^ every year. The annual water use of specific crops is as follows: commodity corn requires 5.938 × 10^6^ m^3^, sunflowers need 0.0623 × 10^6^ m^3^, vegetables use 0.5276 × 10^6^ m^3^, alfalfa requires 5.112 × 10^6^ m^3^, oats consume 0.6237 × 10^6^ m^3^, and silage corn uses 0.5098 × 10^6^ m^3^. The annual water consumption of grain crops is 5.9379 × 10^6^ m^3^, the annual water consumption of economic crops is 0.5899 × 10^6^ m^3^, and the annual water consumption of forage crops is 6.2455 × 10^6^ m^3^.

#### 2.1.2. Analysis of Planting Structure in the Irrigation Area

**Figure 1:**
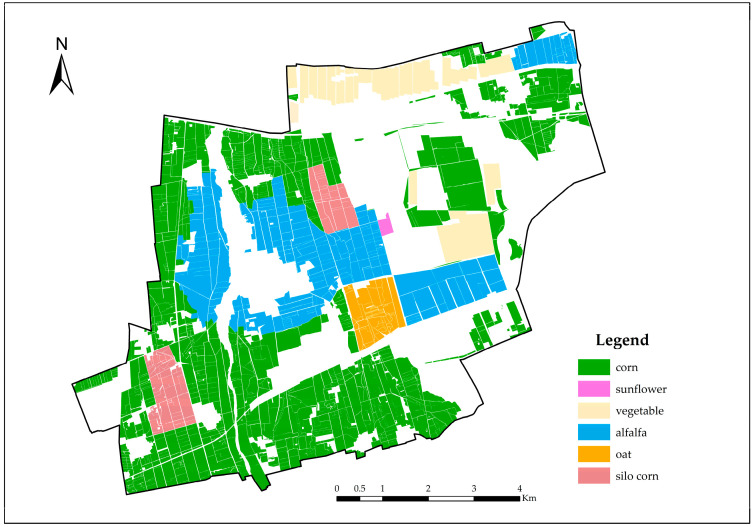
Current situation map of planting structure in the irrigation area.

Utilizing the 91 Satellite Map Assistant software(19.4.0.0) and field survey results, it was determined that crops in the irrigation zone consist of food crops, economic crops, and forage crops. The food crop is corn, while economic crops include vegetables and sunflowers. Forage crops include alfalfa, oats, and silage corn. The land area of the irrigation zone is 71.91 km^2^, with cultivated land covering 43.63 km^2^. The planting area for food crops is 24.72 km^2^, economic crops occupy 4.36 km^2^, and forage crops span 14.55 km^2^. The planting ratio of food to economic to forage crops is 24.72:4.36:14.55. Specifically, corn covers 24.72 km^2^, sunflowers occupy 0.12 km^2^, vegetables span 4.24 km^2^, alfalfa is planted over 10.65 km^2^, oats cover 1.54 km^2^, and silage corn occupies 2.36 km^2^. The current planting structure of the irrigation zone is illustrated in Figure 1.

### 2.2. Evaluation and Analysis of Water Resource Carrying Capacity in the Irrigation Area

#### 2.2.1. Model Result

Using the coefficient of variation method, the weights of various indicators were calculated, as detailed in Table 1.

Based on the computation steps of the gray relational evaluation model, the degrees of association for each subregion were determined, as presented in Table 2.

#### 2.2.2. Evaluation and Analysis of Water Resource Carrying Capacity

According to Table 2, the correlation degree *ε*_3_ for various evaluation standards in the irrigation zone is maximal, indicating that the degree of groundwater resource development and utilization falls under Level III. Overall, the carrying capacity of water resources in the irrigation zone is nearing saturation, and the degree of water resource development and utilization is approaching its limit. Agricultural water consumption in the zone is significant, leading to a gradual decrease in groundwater resources and severe groundwater over-extraction. Among the zones, Sub1 and Sub3 are classified as Level II for groundwater resource development and utilization, indicating that development is at a certain scale, with potential to meet basic agricultural supply and demand needs. In contrast, Sub2, Sub4, Sub5, and Sub6 fall under Level III, indicating substantial development but minimal extraction potential, resulting in severe groundwater over-extraction and significant contradictions between agricultural water supply and demand.

In Sub1 and Sub2, the crop irrigation method for corn is drip irrigation, with a total water resource of 6.0131 × 10^6^ m^3^ and a utilization of 4.9316 × 10^6^ m^3^. In Sub3, the crop irrigation method for corn is furrow irrigation, yielding a total water resource of 1.0544 × 10^6^ m^3^ and a utilization of 0.6358 × 10^6^ m^3^. Thus, the total water resource for food crops is 7.0675 × 10^6^ m^3^, with a utilization of 5.5674 × 10^6^ m^3^, across an irrigation area of 24.72 km^2^. In Sub4, the crop irrigation method for alfalfa is sprinkler irrigation, with a total water resource of 6.0857 × 10^6^ m^3^ and a utilization of 4.386 × 10^6^ m^3^. In Sub5, the crop irrigation method for oats and silage corn is drip irrigation, with a total water resource of 1.3492 × 10^6^ m^3^ and a utilization of 1.1068 × 10^6^ m^3^, resulting in a total water resource for forage crops of 7.435 × 10^6^ m^3^ and a utilization of 5.4928 × 10^6^ m^3^ across an irrigation area of 14.55 km^2^. In Sub6, the crop irrigation method for vegetables is drip irrigation, with a total water resource of 0.7020 × 10^6^ m^3^ and a utilization of 0.5758 × 10^6^ m^3^; thus, the total water resource for economic crops is 0.7020 × 10^6^ m^3^ with a utilization of 0.5899 × 10^6^ m^3^ across an irrigation area of 4.36 km^2^.

#### 2.2.3. Reasons for Water Resource Carrying Capacity Saturation and Planting Structures in Irrigated Areas

In the irrigation zone, the corn (including silage corn) irrigation area is 24.72 km^2^, accounting for 56.7% of the total, utilizing 6.4477 × 10^6^ m^3^, with a demand of 5.268 × 10^6^ m^3^, representing 50.48% of agricultural water usage. Forage water consumption is 6.2455 × 10^6^ m^3^, with a demand of 5.0505 × 10^6^ m^3^, constituting 48.89% of total usage, of which alfalfa accounts for 81.85% of forage water consumption. Economic crop water usage is 0.5899 × 10^6^ m^3^, with a demand of 0.4689 × 10^6^ m^3^, representing 4.62% of total usage. Under current conditions, total water utilization is 12.7733 × 10^6^ m^3^, with total water demand at 10.7874 × 10^6^ m^3^. The water utilization for food crops exceeds that for forage crops, which, in turn, exceeds that for economic crops, with a difference of 1.9859 × 10^6^ m^3^ between total demand and total utilization. During the entire growing season, corn is irrigated six times, requiring an average of 24 m^3^ mu^−1^. Alfalfa is cut four times and irrigated eight times, averaging 40 m^3^ mu^−1^, while oats are cut twice and irrigated six times, averaging 45 m^3^ mu^−1^. The average water usage for economic crops is 20 m^3^ mu^−1^, indicating that forage crops (alfalfa, oats) have a higher average water usage than food crops, which, in turn, have a higher usage than economic crops. However, since alfalfa is not classified as a high-water-consuming crop, the irrigation quotas and frequency in the irrigation zone reveal inefficiencies, leading to water resource wastage. Therefore, there is a need to reduce the average irrigation quota mu^−1^ for crops in the irrigation zone, while corn, as a high-water-consuming crop with a significant planting ratio, requires a rational adjustment of its planting proportion.

Furthermore, the primary irrigation methods for commodity corn and silage corn in the irrigation zone are mostly under-mulch drip irrigation, with a small portion using furrow irrigation. The water resource utilization rate for drip irrigation in the irrigation zone is approximately 82%. However, the integration of under-mulch drip irrigation is low, and farmers often lack a clear irrigation system, leading to inefficient watering practices based on experience. The low recovery rate of plastic mulch results in significant residues, adversely affecting plant absorption of moisture and nutrients, restricting plant growth, and ultimately reducing crop yields, which impacts farmers’ incomes [19]. Losses from furrow irrigation primarily stem from seepage, which constitutes over 80% of total water loss during transmission [20], amounting to approximately 0.2357 × 10^6^ m^3^ per year in the irrigation zone. Thus, the water resource utilization rate for furrow irrigation is about 60%, significantly lower than that of drip irrigation.

Alfalfa irrigation primarily employs sprinkler systems. As a perennial forage crop, alfalfa can be harvested multiple times in a year from a single planting instance. It is irrigated using a rotating sprinkler system, but the height of the sprinkler heads does not align with the varying heights of alfalfa plants at different growth stages. When wind speeds exceed 1.53 to 3.34 m s^−1^, evaporation drift losses can reach 6.27% to 18.54% [21], resulting in a water loss of 0.3205 × 10^6^ m^3^ to 0.9478 × 10^6^ m^3^ per year, leading to an approximate water resource utilization rate of 72% for sprinkler irrigation in the irrigation zone. Oats utilize above-ground drip irrigation, but the drip tapes are prone to aging and clogging issues. The cost of drip tape is approximately 400 CNY mu^−1^, and since oats are harvested twice a year, two installations of drip tape are required, further decreasing farmers’ economic returns.

Therefore, for ensuring food security under conditions of limited water and soil resources, it is crucial to maximize water resource utilization and farmers’ net income. The ratio of food to economic to forage crop planting is an urgent issue that needs resolution.

### 2.3. Optimization Analysis of Planting Structure Under Efficient Utilization of Water Resources

#### 2.3.1. Model Results and Test

Following the solution steps of the genetic algorithm, a program was developed using MATLAB R2016a. After multiple iterations, a set of non-dominated solutions for the model were obtained. The computational results of the model are detailed in Table 3.

The results were validated, and the validation outcomes are presented in Table 4. As shown in the tables, all model results satisfy the specified constraints.

#### 2.3.2. Optimization Analysis of Planting Structure

**Figure 2:**
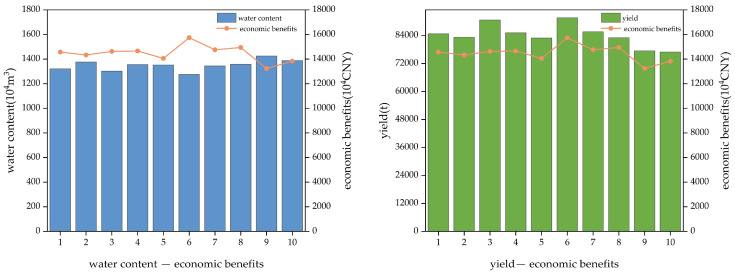
Charts of water content–economic benefits (**left**) and yield–economic benefits (**right**).

A comprehensive analysis of the non-inferior solutions obtained reveals the total yield, total water volume, and total economic benefit, detailed in Figure 2. Among these solutions, the sixth planting ratio yields the minimum total water use, the maximum total yield, and the highest total economic benefit, thus establishing this sixth solution as the “optimal solution” for optimizing the entire industrial structure. As shown in Table 3, under this solution, the area planted with food crops would decrease by 0.0194 × 10^4^ hm^2^, while the area for economic crops would increase by 0.0106 × 10^4^ hm^2^, and the area for forage crops would increase by 0.0116 × 10^4^ hm^2^. The ratio of food to economic to forage crops would be adjusted from 24.72:4.36:14.55 to 23.06:5.18:16.16, with a reduction in food crop area and an increase in both economic and forage crop areas, particularly the latter, which would increase by 1.14 times. The water usage per year would be as follows: corn, 4.9812 × 10^6^ m^3^; sunflowers, 0.3023 × 10^6^ m^3^; vegetables, 0.5723 × 10^6^ m^3^; alfalfa, 5.7962 × 10^6^ m^3^; oats, 0.4520 × 10^6^ m^3^; and silage corn, 0.6403 × 10^6^ m^3^. Water usage would total 4.9812 × 10^6^ m^3^ for food crops, 0.8747 × 10^6^ m^3^ for economic crops, and 6.8885 × 10^6^ m^3^ for forage crops. According to Figure 2, the total economic benefit would reach 15.08 million CNY, an increase of 655.92 thousand CNY compared to the original planting ratio; the total water utilization per year would be 12.7444 × 10^6^ m^3^, reduced by 0.0289 × 10^6^ m^3^ compared to the original ratio. The economic benefit generated per cubic meter of utilized water would be 13.35 CNY, an increase of 0.5 CNY compared to the original economic benefit; the total yield would reach 91,556 tons, an increase of 4340.86 tons from the original planting ratio, with the economic benefit per kilogram of yield at 1.72 CNY, an increase of 0.1 CNY from the original benefit.

**Figure 3:**
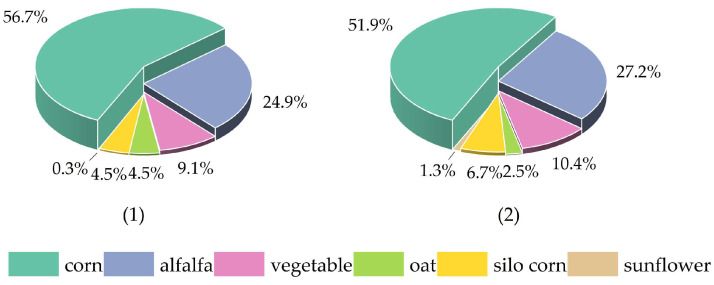
Before (**1**) and after (**2**) the optimization and adjustment of the planting structure in the irrigation area.

The changes in crop planting ratios before and after optimization are depicted in Figure 3. It is evident that the planting ratio of corn has been adjusted from 56.63% to 51.94%; the planting ratio of sunflowers has increased from 0.27% to 1.31%; the planting ratio of vegetables has increased from 9.07% to 10.36%; the planting ratio of alfalfa has increased from 24.93% to 27.20%; the planting ratio of oats has decreased from 4.53% to 2.51%; and the planting ratio of silage corn has increased from 4.53% to 6.68%.

## 3. Discussion

This study employed a gray relational analysis model to evaluate the groundwater resource carrying capacity in the mixed-cropping irrigation zones of the Yellow River Basin in Inner Mongolia for 2023. It subsequently analyzed the reasons for the saturation of the groundwater resource carrying capacity in these zones and identified issues related to the cropping structure. Finally, the study optimized the cropping structure based on a multi-objective genetic algorithm. Zuo [22] indicated that the water resource carrying capacity in Inner Mongolia is classified as Level III (critical), which is below the saturated carrying capacity observed in this study. This discrepancy may stem from Zuo’s analysis encompassing the entire Inner Mongolia Autonomous Region, while this study focused solely on a smaller area within the region and exclusively examined groundwater resources, leading to certain deviations. Zhang [23] demonstrated that, in the Inner Mongolia section of the Yellow River Basin, excluding Alxa League, the degree of groundwater development and utilization is extremely high, with agricultural irrigation accounting for 72.5% of total water usage. In this study, the degree of groundwater development and utilization was approximately 84%, with agricultural irrigation water use constituting a remarkable 84% of the total usage. While the development and utilization levels were consistent with Zhang Ningning’s findings, the proportion of agricultural irrigation water use was higher, likely due to certain areas within the irrigation zone being over-extracted groundwater zones [24]. Additionally, the insufficient introduction of water from the Yellow River and unclear irrigation practices for corn led to substantial waste in agricultural water usage in our study. M. Hu [25] indicated that the average carrying capacity in Inner Mongolia is 0.25 (on a scale where 1.0 is the maximum), and at the basin scale, the carrying capacity of water resources in the Inner Mongolia Yellow River Basin is the lowest, at 0.17. Kang [26] further noted that, while agricultural water resources in Inner Mongolia are not overloaded, their carrying potential is limited. These findings reinforce the conclusion of this study regarding the saturation of water resource carrying capacity and the low efficiency of water utilization in the irrigation zone. Ma [27] utilized a multi-objective genetic algorithm in Manas County to suggest increasing the planting proportion of corn while reducing that of vegetables. In contrast, this study recommends a decrease in the proportion of corn and an increase in vegetables, reflecting the high water consumption associated with corn and the greater economic benefits derived from vegetable cultivation. Regional differences necessitate varying developmental strategies. The proposed cropping structure aims to balance food production with water conservation [28]. Although the area planted with food crops would diminish in our model, increasing the yield per unit area could ensure food security. The area for forage crops (alfalfa and oats) has expanded in our proposal; however, the average water usage per mu and the frequency of irrigation for these crops are significant. The average water requirement for alfalfa is approximately 40 m^3^ mu^−1^ (599 m^3^ hm^−2^). Liang [29] reported that the average water usage for alfalfa ranges between 300 and 375 m^3^ hm^−2^, indicating that the average water requirement in this study is significantly higher than in Liang’s findings. This suggests that, despite the use of water-saving technologies, there remains a notable wastage of water resources. Consequently, there is a need to further optimize the irrigation system and improve water-saving technology and infrastructure to reduce water losses.

This study explored the evaluation of groundwater resource carrying capacity applied to agricultural irrigation in a specific irrigation zone and optimized the cropping structure. However, it lacked a comprehensive analysis of the adjustments and optimizations to the cropping structure following the enhancement of irrigation practices and the optimal allocation of water resources. Future work will focus on optimizing the irrigation practices, readjusting cropping structure ratios, and determining the optimal water usage plan based on evaluations of water resource carrying capacity and cropping structure optimization, while integrating the growth and water consumption patterns of crops in the irrigation zone to ensure rational water usage among food, economic, and forage crops.

## 4. Materials and Methods

### 4.1. Region Description

**Figure 4:**
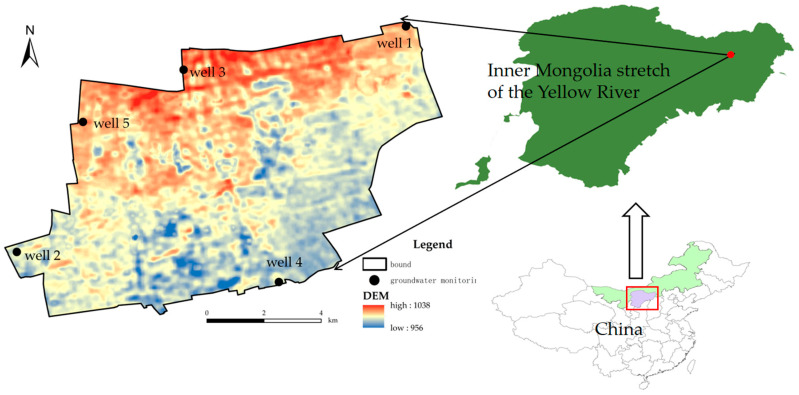
Overview of the study area.

The Inner Mongolia segment of the Yellow River is located at the northernmost point of the river’s ‘J’-shaped bend (37°35′–41°50′ N, 106°10′–112°50′ E). It has a total available conventional water resource of 5.86 billion m^3^, which is less than half of the national average, making it one of the most water-scarce regions in the Yellow River Basin. The Inner Mongolia Yellow River Basin is home to major domestic dairy companies, including Yili, Mengniu, and Shengmu, which have a substantial demand for artificial forage. The study area is located on the northern bank of the Yellow River at the southern foot of the Daqingshan Mountains, specifically in the Tumu Chuan Plain in the Beishazhou Township and Bikeqi Town of Tumoteyouqi (40°38′~40°44′ N, 111°16′~111°24′ E), covering a total land area of 108,000 mu (approximately 7200 hectares) (Figure 4). This region falls within a continental arid climate zone, with an average annual precipitation of 403.80 mm, predominantly concentrated in the summer months of June to August. The average annual evaporation is 1870.30 mm, the average annual temperature is 7.3 °C, and the total annual sunshine duration averages 2876.5 h, with a relatively short frost-free period of about 133 days. The main crops cultivated include corn, vegetables, alfalfa, oats, and silage corn. Irrigation in the study area utilizes both surface water and groundwater, primarily employing drip irrigation, sprinkler irrigation, and furrow irrigation methods.

### 4.2. Experimental Design and Data Collection

#### 4.2.1. Groundwater Depth

**Figure 5:**
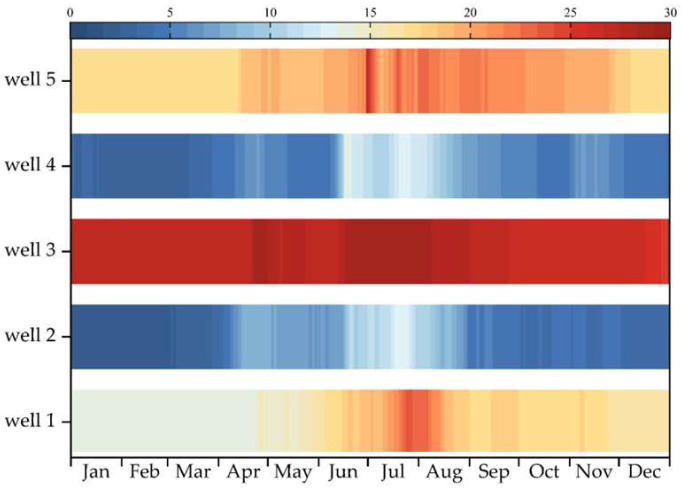
Map of groundwater depth changes in 2022.

Groundwater in the area is primarily sourced from deep wells, with a total of five groundwater monitoring wells. Figure 5 shows the groundwater depths for these five monitoring wells in 2023. According to the annual average, the groundwater depth ranks as follows: Well 3 > Well 5 > Well 1 > Well 4 > Well 2. Well 1 has an average groundwater depth ranging from 13.71 to 21.01 m, Well 2 from 1.95 to 11.76 m, Well 3 from 25.80 to 28.78 m, Well 4 from 3.32 to 11.56 m, and Well 5 from 17.01 to 21.98 m. The lowest groundwater depths are observed during July and August, coinciding with peak irrigation water usage for crops. The data were sourced from national monitoring well statistics.

#### 4.2.2. Meteorological Data Acquisition

**Figure 6:**
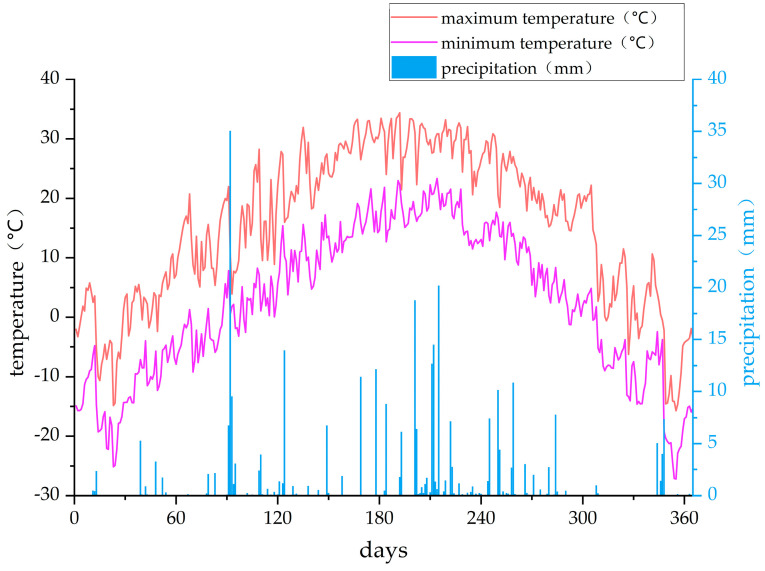
Precipitation and temperature changes in 2023.

The maximum and minimum temperature and precipitation data for 2023 are illustrated in Figure 6. The total annual precipitation for 2023 was 320.81 mm, primarily occurring between July and September. The temperatures in 2023 ranged from −27.21 °C to 34.41 °C, with an average temperature variation between 2.51 °C and 15.43 °C, yielding an overall average of 8.91 °C. The monthly average maximum temperature reached 29.94 °C in July, while the monthly average minimum temperature was −15.74 °C in January. Meteorological data were obtained from the Tumoteyouqi Meteorological Station.

#### 4.2.3. Subdivision of Irrigation Area

**Figure 7:**
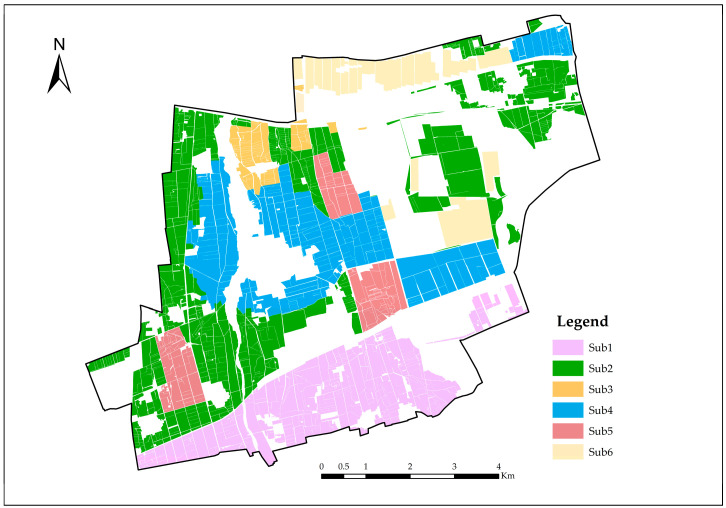
Sub-basin agricultural land zoning map.

The zoning map of the study area (Figure 7) was utilized for the evaluation and analysis of water resource carrying capacity. The study area was primarily characterized by irrigated silt and saline soils, categorized into three types of crops: food crops, forage crops, and economic crops. The irrigation methods employed included sprinkler irrigation, drip irrigation, and furrow irrigation. The specific zoning is illustrated in Figure 4: Sub1 represents irrigated silt with food crops and drip irrigation; Sub2 denotes saline soil with food crops and drip irrigation; Sub3 includes saline soil with food crops and furrow irrigation; Sub4 consists of saline soil with forage crops and sprinkler irrigation; Sub5 features saline soil with forage crops and drip irrigation; and Sub6 indicates saline soil with economic crops and drip irrigation.

### 4.3. Construction of Comprehensive Evaluation Model of Water Resource Carrying Capacity

#### 4.3.1. Index System Construction

The carrying capacity of water resources in the irrigation area is influenced by multiple factors. Direct factors include aspects related to water supply and demand, while indirect factors encompass industrial structure and economic development levels. Referring to the national water resource evaluation index system and standards, and taking into account the variability in water resources and different methods of utilization, five main evaluation factors were selected for the comprehensive assessment of water resource carrying capacity: water resource utilization rate, irrigation rate of cultivated land, degree of water resource development and utilization, supply modulus, and demand modulus [30,31,32]. The meanings of these indicators are as follows (Table 5):

(1) Water resource utilization rate (X_1_ ): utilized water volume/annual water resource supply volume (%);
(2) Irrigation rate of cultivated land (X_2_): irrigated area/total land area (%);
(3) Degree of water resource development and utilization (X_3_): water supply volume/total water resources (%);
(4) Supply modulus (X_4_): annual water supply volume/total land area (10^4^ m^3^/km^2^);
(5) Demand modulus (X_5_): current annual water demand/total land area (10^4^ m^3^/km^2^).

#### 4.3.2. Weight Determination

Based on these five indicators, the characteristic values for water resource indicators in the study area were established, as detailed in Table 1. When determining the weights of the evaluation indicators, the authors preferred using subjective judgment methods, such as the analytic hierarchy process (AHP). However, this approach may lead to evaluation results that are influenced by personal biases. In contrast, the coefficient of variation method determines indicator weights by analyzing the data, thereby reflecting the variability of the indicators more objectively, representing a more objective method for weight determination. In this study, the weights of the indicators were calculated using the coefficient of variation method, with specific calculation steps outlined in References [33,34,35].

#### 4.3.3. Gray Correlation Analysis Model

The gray relational evaluation system determines the rank of a reference sequence by comparing its degree of association with various standard evaluation sequences. This model consists of six zones, which serve as reference sequences, while the evaluation criteria are treated as comparison sequences. The obtained measurement data were normalized to compute correlation coefficients and degrees of association. The analysis of association essentially examined the spatial geometric relationships between comparative sequence data, obtaining the maximum association value to establish the rank of the reference sequence. Subsequently, by comparing the reference sequence with the comparison sequences (standard sequences) and their respective maximum association values, a ranking of the reference sequences was achieved, enabling classification of the zones. The computational methods and detailed steps of the model can be found in References [36,37,38].

#### 4.3.4. Evaluation Grade Division

Based on the existing literature [39,40,41,42] and in consideration of the actual conditions in the irrigation area, the aforementioned factors were classified into three levels of influence on groundwater resource carrying capacity: I, II, and III. Level I indicated good groundwater resource conditions, suggesting that the area still possessed significant carrying capacity and potential for development, with agriculture currently in a low-efficiency water use phase. Level II signified average groundwater resource conditions, indicating that groundwater resources had reached a certain level of development and utilization, yet still had further development potential, with agriculture transitioning from a low-efficiency water use phase to a high-efficiency phase. Level III reflected poor groundwater resource conditions, suggesting that the capacity was gradually approaching threshold limits, with agriculture currently in a high-efficiency water use phase; if this were to continue, it would lead to water resource shortages and groundwater over-extraction, constraining socio-economic development. Table 6 presents the classification of water resource evaluation indicators for the study area.

### 4.4. Planting Structure Optimization Model

#### 4.4.1. Model Construction

This study employed a multi-objective genetic algorithm to solve the crop structure optimization model, ensuring that the optimization plan minimized agricultural water usage while maximizing economic benefits. The crop structure optimization model began with mathematical modeling. The first step was to determine the decision variables for optimization, with the model utilizing the planting areas of different crops as the decision variables, totaling six decision variables. The second step involved establishing the objective functions for optimization, selecting minimum water usage and maximum economic benefit as the targets. The third step set the necessary constraints, primarily related to cultivated land area, food security, and crop yield. The fourth step identified the parameters required for the model. Detailed steps for calculating the crop structure optimization model can be found in References [43,44].

#### 4.4.2. Objective Functions

1. The water consumption target was calculated as follows:
  (1) $$minQ_{i}=\sum_{j=1}^{m}q_{j}^{\left(i\right)}\cdot s_{1j}^{\left(i\right)}$$
  where Qi represents the total agricultural water consumption in year i of the planning horizon, measured in ten thousand cubic meters (10,000 m^3^); $q_{j}^{\left(i\right)}$ denotes the gross irrigation quota for crop j in year i, expressed in cubic meters per hectare (m^3^/hm^2^); and $s_{1j}^{\left(i\right)}$ indicates the planting area of crop j in year i, also in ten thousand hectares (10,000 hm^2^).
2. The economic benefit target was calculated as follows:
  (2) $$maxY_{i}=\sum_{j=1}^{m}p_{j}^{\left(i\right)}\cdot y_{j}^{\left(i\right)}\cdot s_{1_{j}}^{\left(i\right)}$$
  where Yi is the total economic benefit of agricultural and pastoral planting structures in year i, measured in ten thousand CNY; $p_{j}^{\left(i\right)}$ is the unit price of product j in year i, expressed in CNY per kilogram (CNY/kg); and $y_{j}^{\left(i\right)}$ is the yield per unit area of crop j in year i, measured in kilograms per hectare (kg/hm^2^).

#### 4.4.3. Constraint Conditions

1. The cultivated area was calculated as
  (3) $$\sum_{j=1}^{m}s_{1_{j}}^{\left(i\right)}\leq \alpha^{\left(i\right)}\cdot A_{g}^{\left(i\right)}$$
  where α(i) represents the cropping index in year i, and $A_{g}^{\left(i\right)}$ signifies the arable land area in year i, measured in ten thousand hectares (10,000 hm^2^).
2. Food security was calculated as
  (4) $$\sum_{j=1}^{m}y_{j}^{\left(i\right)}\cdot s_{1_{j}}^{\left(i\right)}\geq P^{\left(i\right)}$$
  where P(i) denotes the grain demand in year i, expressed in ten thousand tons (10,000 t).
3. Crop yield was calculated using the following equation:
  (5) $$y_{j}^{\left(i\right)}\cdot s_{1_{j}}^{\left(i\right)}\geq P_{j}^{\left(i\right)}$$
  where P^(i)^ denotes the grain demand in year i, expressed in ten thousand tons (10,000 t).
4. The equilibrium condition was calculated as
  (6) $$s_{1_{j}}^{\left(i\right)}\geq A_{{1j}_{min}}^{\left(i\right)}$$
  where the minimum required planting area for crop j in year i is represented by $A_{1jmin}^{\left(i\right)}$, in ten thousand hectares (10,000 hm^2^).
5. The non-negative condition was
  (7) $$s_{1_{j}}^{\left(i\right)}\geq 0$$

#### 4.4.4. Decision Variables

The crops in the study area were categorized into food crops, economic crops, and forage crops, including corn, sunflowers, vegetables, alfalfa, oats, and silage corn. The planting area of each crop was treated as a decision variable, with detailed descriptions of each decision variable provided in Table 7.

#### 4.4.5. Model Parameter Determination

The model parameters were determined mainly according to the *Hohhot Statistical Yearbook* (2023) [45] and field investigation, and the planning year was i = 2030. The details were as follows:

1. Multiple cropping index: $\alpha^{\left(i\right)}=1.02$
2. Cultivated area: $A_{g}^{\left(2023\right)}=0.44\times 10^{4}$ hm; $A_{g}^{\left(2030\right)}=0.44\times 10^{4}\;{hm}^{2}$
3. Minimum corn planting area:$\;A_{11}^{\left(i\right)}=0.20\times {10^{4}\;hm}^{2}$
4. Crop yield

The estimation of crop yields was primarily based on the sowing area and production data from the *Statistical Yearbook of Hohhot* (2023), as well as field surveys and historical crop yield data. Detailed information can be found in Table 8.

5. Crop irrigation quota

The irrigation quotas for each crop were primarily determined based on the “Standards for Industry Water Quotas in Inner Mongolia” [46] and previous crop irrigation data, as detailed in Table 9.

6. Unit price of crops

The unit price data for major crops was sourced from the “Compilation of National Agricultural Product Cost and Benefit Data” (2023) [47], which included information on the costs and returns of agricultural products and market surveys, as detailed in Table 10.

### 4.5. Data Source

This study drew data from several sources, including the *2023 Hohhot Statistical Yearbook*, the Inner Mongolia Industry Water Quota Standard (DB15/T 385-2020), the 2023 Compilation of National Agricultural Product Cost and Benefit Data, the first half of 2023 progress report and work plan from the Tumotey Right Banner Water Affairs Bureau, and the 2019 Technical Report on the Third National Water Resource Survey and Evaluation in the Inner Mongolia Autonomous Region, along with field survey results.

## 5. Conclusions

This study established a water resource carrying capacity evaluation model based on gray relational analysis and an optimization model for a cropping structure based on machine learning. It investigated the water resource availability and cropping structure in mixed-cropping irrigation zones for food, economic, and forage crops. The main conclusions were the following:

1. The degree of groundwater resource development and utilization in the irrigation zone was rated overall at Level III, indicating that the water resource carrying capacity is approaching saturation. Agricultural water consumption was substantial, leading to a gradual decrease in groundwater resources and serious over-extraction issues. Specifically, Sub1 (loamy soil, food crops, and drip irrigation) and Sub3 (saline soil, food crops, and furrow irrigation) were rated at Level II, demonstrating potential to meet basic agricultural supply and demand. In contrast, Sub2 (saline soil, food crops, and drip irrigation), Sub4 (saline soil, forage crops, and spray irrigation), Sub5 (saline soil, forage crops, and drip irrigation), and Sub6 (saline soil, economic crops, and drip irrigation) were rated at Level III, indicating substantial development but limited extraction potential, with pronounced supply–demand conflicts.
2. The proportion of corn (including silage corn) in the irrigation zone was as high as 56.7%, resulting in an overall low water resource utilization efficiency. The total water utilization amounts were as follows: food crops (5.5674 × 10^6^ m^3^/a) > forage crops (5.4928 × 10^6^ m^3^/a) > economic crops (0.5899 × 10^6^ m^3^/a). The average water utilization per mu was highest for forage crops (alfalfa: 40 m^3^ mu^−1^, oats: 45 m^3^ mu^−1^), followed by food crops (24 m^3^ mu^−1^) and economic crops (20 m^3^ mu^−1^). The trend of water resource wastage based on irrigation methods was as follows: furrow irrigation > spray irrigation > drip irrigation. It is essential to rationally adjust cropping proportions, reduce per mu irrigation quotas, and improve irrigation methods to enhance overall water resource utilization efficiency and farmers’ economic benefits.
3. To maximize the economic benefits and water resource utilization rates, it is recommended to reduce the planting area of food crops by 0.0194 × 10^4^ hm^2^, while increasing the area for economic crops by 0.0106 × 10^4^ hm^2^ and the area for forage crops by 0.0116 × 10^4^ hm^2^. Consequently, the cropping ratio of food, economic, and forage crops would be adjusted from 24.72:4.36:14.55 to 23.06:5.18:16.16. This adjustment would lead to a total water utilization reduction of 0.0289 × 10^6^ m^3^ per year, an increase in total yield of 4340.86 tons, and an increase in total economic benefit of 6,559,200 CNY, thus leading the cropping structure towards greater rationality.

## Figures and Tables

**Table 1:** Weight of each indicator.

	X_1_	X_2_	X_3_	X_4_	X_5_
Weight	0.1549	0.1477	0.2575	0.2318	0.2081

**Table 2:** Calculation result of correlation degree of the irrigation area.

Partition		Sub1	Sub2	Sub3	Sub4	Sub5	Sub6
**Gray correlation degree**	$\varepsilon_{1}$	**0.4705**	**0.4980**	**0.5737**	**0.5422**	**0.5237**	**0.5188**
	$\varepsilon_{2}$	**0.7851**	**0.6879**	**0.7481**	**0.7266**	**0.7562**	**0.6569**
	$\varepsilon_{3}$	**0.7850**	**0.7389**	**0.7083**	**0.7267**	**0.7567**	**0.6645**
**Grade**		**II**	**III**	**II**	**III**	**III**	**III**

**Table 3:** Non-inferior solutions of the optimal model of agricultural structure in the irrigated area.

Non-Inferior Solutions	Optimization Variables					
	S_11_	S_12_	S_13_	S_14_	S_15_	S_16_
1	0.2194	0.0012	0.0300	0.1239	0.0354	0.0301
2	0.2238	0.0011	0.0232	0.1308	0.0407	0.0301
3	0.2256	0.0043	0.0320	0.1115	0.0324	0.0396
4	0.2238	0.0041	0.0290	0.1308	0.0300	0.0300
5	0.2326	0.0017	0.0222	0.1208	0.0417	0.0300
6	0.2356	0.0058	0.0460	0.1208	0.0112	0.0296
7	0.2238	0.0017	0.0300	0.1308	0.0300	0.0300
8	0.2195	0.0041	0.0322	0.1308	0.0354	0.0239
9	0.2208	0.0063	0.0105	0.1408	0.0400	0.0301
10	0.2343	0.0042	0.0189	0.1308	0.0405	0.0205

**Table 4:** Verification of model calculation results.

Serial Number	Item	Calculated Value	Constraints	
1	Arable land area	0.44	Less than	0.45
2	Food security	11.60	Greater than	10.74
3	Crop yield: corn	34.71	Greater than	29.85
4	Equilibrium conditions	Satisfaction	Greater than	The minimum acreage
5	Non-negative conditions	Satisfaction	Greater than	0

**Table 5:** Characteristic value of water resources index in the study area.

Evaluation Index	Sub1	Sub2	Sub3	Sub4	Sub5	Sub6
X_1_	82.01	82.02	60.30	72.07	82.03	82.02
X_2_	81.04	81.60	54.29	84.81	92.86	81.56
X_3_	84.02	84.02	84.00	84.01	84.03	84.00
X_4_	17.50	8.98	36.15	35.96	26.99	5.69
X_5_	16.64	8.17	22.58	28.71	23.04	4.53

**Table 6:** Water resources evaluation index of the study area.

Grade	X_1_	X_2_	X_3_	X_4_	X_5_
I	<30	<40	<50	<10	<10
II	30~60	40~80	50~75	10~15	10~15
III	>60	>80	>75	>15	>15

**Table 7:** Crop decision variables in the study area.

Industry	Crop	Decision Variable	Unit	Quantity
Grain crops	Corn	S_11_	10^4^ hm^2^	0.25
Cash crops	Sunflower	S_12_	10^4^ hm^2^	0.0012
	Vegetable	S_13_	10^4^ hm^2^	0.04
Forage crops	Alfalfa	S_14_	10^4^ hm^2^	0.11
	Oat	S_15_	10^4^ hm^2^	0.02
	Silo corn	S_16_	10^4^ hm^2^	0.02

**Table 8:** Crop yield in the study area.

Crop	Corn	Sunflowers	Vegetables	Alfalfa	Oats	Silage Corn
Yield (kg hm^−2^)	14,925	2048	51,608	11,940	10,447	59,701

**Table 9:** Crop irrigation quota in the study area.

Crop	Corn	Sunflowers	Vegetables	Alfalfa	Oats	Silage Corn
Irrigation norm (m^3^ hm^−2^)	2158	2340	1244	4797	4047	2158

**Table 10:** Unit price of crops in the study area.

Crop	Corn	Sunflowers	Vegetables	Alfalfa	Oats	Silage Corn
Price (CNY kg^−1^)	1.4	3.50	2.17	3.2	3.00	0.43

## Data Availability

The data are contained within this article.
